# Measuring the influence of perceived organizational support, organizational hierarchy culture, strategic human resource management, and psychological empowerment on the return to work

**DOI:** 10.3389/fresc.2025.1679281

**Published:** 2026-01-02

**Authors:** Nadra Tawfig, Suhad Farsi, Alaa Lary, Kholod Aggad

**Affiliations:** College of Business Administration, University of Business and Technology, Jeddah, Saudi Arabia

**Keywords:** perceived organizational support, organizational hierarchy culture, strategic human resource management, psychology empowerment, return to work

## Abstract

In this era of high competition and pandemic, the return to work is challenging because it requires organizations to balance operational demands with individualized support for employees recovering from psychological, physical, and/or personal setbacks. Additionally, managerial expectations, misalignment between workplace culture, and employee readiness can hinder effective reintegration, leading to reduced relapse or productivity. This study aimed to examine the influence of key organizational and psychological factors on employees' return-to-work. A stratified sampling approach was employed to collect data from 370 employees who had successfully returned to work after experiencing work-related injuries and diseases. Eight hypotheses were formulated and validated using structural equation modeling with SmartPLS. Findings from the present study reveal that perceived organizational support has an insignificant impact on the return to work; however, it has a significant impact on strategic human resource management and psychological empowerment. In addition, the present study confirmed a significant impact of organizational hierarchy culture on return-to-work and psychological empowerment. Thus, strategic human resource management had a significant impact on return-to-work rates and psychological empowerment. Finally, psychological empowerment had a significant impact on the return to work. The study highlights the importance of culturally aligned human resource strategies and psychological factors in fostering successful employee reintegration after work absence. These findings have practical implications for human resource professionals and policymakers aiming to design effective, inclusive, and sustainable return-to-work programs tailored to hierarchical organizational environments.

## 1 Introduction

Return to Work (RTW) refers to the processes and policies organizations implement to support employees in resuming their job responsibilities after a prolonged absence due to medical, psychological, or personal reasons (1, 2). It encompasses a wide range of harmonized efforts to ensure that employees are effectively, safely, and sustainably reintegrated into the workplace. Franche & Krause (3) further outlined that the RTW processes often include phased returns, modified job duties, workplace accommodations, medical clearances, and ongoing monitoring to facilitate a smooth transition. Moreover, RTW can be broadly defined as a set of measurement and support activities designed to help employees with disabilities or illnesses return to work after undergoing occupational reintegration treatment for their conditions (4, 5). Returning to work after recovering from an illness, stroke, or disability is crucial from both societal and individual perspectives (6). Work limitations can lead to reduced income, financial hardship, and a lower quality of life (66). “The World Health Organization's International Classification of Functioning, Disability, and Health (ICF)” is an integrative, biopsychosocial model that illustrates the correlation between the individual and their working or living environmental context as a contributing factor of disease/disability (67).

Existing empirical studies suggest that organizational support plays a crucial role in shaping employees' RTW experiences. For instance, Park et al. (7) found that support from the organization and hierarchical cultures offer structure and procedural clarity, which are particularly beneficial in reducing reintegration anxiety. Jeong et al. (8) stated that several employees who are sick still fail to maintain a quality of life and work stability after the stroke, which creates a negative perspective in the minds of the organizational hierarchy. However, top management in an organization typically holds its views and performs its job responsibilities in accordance with its position. Different opinions about the patient's stroke and other beliefs regarding qualified therapies influence stakeholders' support for the patient's return to work based on job description criteria (9, 10). Another study by Baril et al. (11) found that organizational hierarchy is associated with greater affective commitment and psychological resilience, both of which are crucial to a successful RTW process. On the other hand, psychological empowerment, characterized by feelings of autonomy, competence, and impact, has also received considerable attention in recent literature. According to Drazic et al. (12), empowered employees are more proactive and adaptive, enabling them to navigate return-to-work challenges more effectively. This transition can be complex and challenging, involving physical, emotional, and logistical aspects. Additionally, SHRM—the alignment of HR practices with long-term strategic goals—has been identified as a key facilitator of organizational agility and employee engagement during reintegration processes (13). In this view, Philpot and Gavrilova Aguilar (14) observed that the HR department provides support, resources, and guidance to employees with disabilities as they navigate the RTW journey. By addressing physical and emotional needs and implementing workplace accommodations, these entities contribute to the successful and sustainable reintegration of employees with disabilities and/or illnesses into the workforce (15).

Empirically, Cavanagh et al. (16) found that in several developing countries, primarily in Asia, HR departments in public and private organizations do not offer exceptional return-to-work policies for employees who are disabled or sick. On the other hand, Bastien & Corbière (17) found that HR departments in developing countries offer reliable work policies and protocols in both public and private organizations, which motivate employees to RTW after completing treatment. Although less likely, HRM in some countries has developed limited policies and protocols to support returning-to-work employees, particularly in developing countries (18, 19). Similarly, in Saudi Arabia, RTW policies are not widely discussed, including standard working environments, working hours, and health incentives. In which most disabled or ill employees are not able to RTW happily. Accordingly, rehabilitation centers also do not educate and motivate psychologically disabled or sick employees of the RTW. Yet, in public and private organizations, policies regarding RTW for survivors are not widely discussed (20); in fact, few organizations are considering bringing survivors back to work (21). Organizations that do not have explicit RTW policies for survivors and have inferior communication standards with their survivors' employees (22). On the other hand, prior empirical studies have shown that in developed countries, employees who are sick or disabled are directly concerned with organizational support in finding suitable ways to maintain stability in their quality of life and job environment (23). The job and the environment are evaluated based on the impact of cognitive, physical, emotional, organizational, economic, and social factors on the job conditions (24). Furthermore, for several patients, occupation plays a significant societal role, serving as a source of livelihood, while work limitations can have minimal impact on their social life. The return-to-work process for an employee after diagnosis is a multidisciplinary endeavor involving multiple stakeholders from the employer's perspective (68). Additionally, legislative institutions have designed return-to-work protocols for both public and private institutions in a country (69). The legislation also plays a dynamic role in facilitating employees' return to work before or after treatment, as noted by De Rijk et al. (70). Rejoining a job helps the employee maintain their quality of life and working ability. Notably, the RTW is likely to depend on organizational support, the environment, HR departments, and employees' psychological readiness and perceived support.

The present study aims to examine the impact of four core constructs—Organizational Hierarchy Culture (OHC), Perceived Organizational Support (POS), Psychological Empowerment (PE), and Strategic Human Resource Management (SHRM)—on RTW outcomes, employing conservation of resources theory and social exchange theory. OHC reflects the structural, rule-based orientation of organizations; POS captures employees' perceptions of being valued and supported; PE addresses individuals' sense of competence and autonomy in their roles; and SHRM encompasses strategic practices designed to align human capital with organizational goals. In practice, the present study focused on the employees who had been absent from work due to illness and/or injury. Prior studies suggested that the conservation of resources theory and social exchange theory help predict the relationship between organizational practices and employee outcomes (25, 26). Thus, the present study extended the theoretical underpinning by proposing psychological empowerment as a mediating variable, in which employees' perceptions of competence, control, and autonomy influence their job performance. Moreover, the present study examines how psychological empowerment operates in a high-power-distance culture, leveraging hierarchical structures and power differences, to understand its impact on employee performance. The framework contributes to an understanding of its mediation mechanism and its organizational implications across different cultural settings.

Despite this growing body of research, several gaps remain unaddressed. First, most studies investigate these constructs in isolation rather than within an integrated framework that captures their interdependencies. Second, limited attention has been paid to SHRM's broad role in translating cultural and psychological dynamics into RTW outcomes. SHRM plays a wide role in designing and implementing organizational goals, enhancing performance, and sustaining the competitive advantage, aligning with overall business strategies (15). In addition, SHRM focuses on integrating workforce planning, talent development, long-term planning, performance management, and organizational culture to achieve the company's objectives and drive business success. Third, empirical investigations into these relationships remain scarce in Arab countries, where contextual factors such as rigid hierarchies and resource constraints may affect RTW implementation.

## 2 Literature review and hypotheses development

### 2.1 Perceived organizational support, return to work, strategic human resource management, and psychological empowerment

Perceived organizational support refers to employees' belief that their organization values their contributions to their well-being and productivity (27). When employees perceive greater organizational support—such as communication, accommodations, reintegration programs, and emotional support—they are more likely to feel confident and secure in resuming work after an absence, particularly due to mental and/or physical illness (28). This psychological safety fosters a positive commitment, attitude, and a willingness to reciprocate through enhanced work engagement and effort upon RTW (29). Several past empirical studies have documented a significant, positive relationship among perceived organizational support, RTW, strategic human resource management (SHRM), and psychological empowerment. For instance, Shaw et al. (30) found that employees with high perceived organizational support reported smoother transitions back to work, particularly when organizational support was evident in both practice and policy. Accordingly, Aljabri et al. (31) stated that employees recovering from occupational injuries were more likely to RTW earlier when they perceived a strong support system within their organization. However, in the context of RTW, SHRM practices such as flexible work arrangements, health and safety policies, continuous dialogue with healthcare providers, and reintegration programs are designed to enhance employee well-being and sustain workforce productivity (32). SHRM treats RTW not as a reactive function but as a proactive strategy embedded in workforce planning. While both perceived organizational support and SHRM have been investigated separately in relation to RTW, there is limited empirical research linking these constructs directly to RTW within a unified model, particularly in developing countries or in high-stress sectors such as healthcare and manufacturing. Moreover, Suijkerbuijk et al. (33) noted that perceived organizational support enhances psychological empowerment and motivation, which are crucial to successful RTW outcomes. Thus, limited past studies examined and concluded that there is a direct relationship between perceived organizational support and psychological empowerment in the context of RTW. Based on the above arguments, the present study proposes the following hypotheses.

H1: Perceived organizational support has a significant positive impact on the return to work

H2: Perceived organizational support has a significant and positive impact on strategic human resource management

H3: Perceived organizational support has a significant and positive impact on psychological empowerment

### 2.2 Organizational hierarchy culture, psychological empowerment, and return to work

Organizational hierarchy culture refers to a well-ordered, control-oriented organizational environment characterized by high power distance, rule-bound procedures, centralized decision-making, and a focus on stability and efficiency (1, 8, 34). In such organizational cultures, employees are often productive within rigid protocols and clearly defined roles, which can both facilitate and hinder RTW processes. Moreover, Jansen et al. (35) noted that a strong hierarchy can support RTW by establishing clear reintegration procedures, complying with legal standards, and maintaining consistency in the treatment of returning employees. Excessive rigidity may reduce flexibility, autonomy, and psychological safety—factors crucial to successful reintegration, especially for employees recovering from illness or injury. Existing studies reported mixed outcomes. For instance, Yuan et al. (36) examined and concluded that structured RTW protocols in hierarchical firms led to higher compliance and smoother administrative processing. However, Santos et al. (37) demonstrated that overly bureaucratic environments may delay RTW by discouraging open communication and personalized adjustments. In addition, Hamzah et al. (71) suggested that hierarchy-oriented cultures should adapt their formal policies to be more inclusive and employee-centric, thereby improving RTW success rates. For instance, in hierarchical cultures, the success of RTW depends on leadership support, top-down endorsement, and gradual alignment with existing power structures, even though adapting formal policies to be more employee-centric and inclusive may enhance their effectiveness (1).

On the other hand, flatter and more participative cultures were shown to enhance employees' sense of competence and meaning at work. In this study, Turner (38) found that employee empowerment initiatives are less effective in highly hierarchical organizations because decision-making authority remains reserved for upper management, thereby reducing employees' self-efficacy and perceived control. These patterns suggest that the more an organization relies on hierarchical structures, the more it may undermine employees' psychological empowerment practically in the context of RTW. Therefore, existing studies have not examined or concluded the impact of organizational hierarchy culture on the psychological empowerment of RTW employees, particularly in high-power-distance countries such as Saudi Arabia. Thus, the following hypotheses were proposed.

H4: Organizational hierarchy culture has a significant and positive impact on the return to work

H5: Organizational hierarchy culture has a significant and positive impact on psychological empowerment

### 2.3 Strategic human resource management, return to work, and psychological empowerment

SHRM refers to the proactive alignment of human capital strategies with organizational objectives to enhance long-term performance and workforce sustainability (15). Unlike traditional HRM, which often focuses on routine administrative tasks, SHRM adopts a forward-looking approach that integrates policies related to employee development, well-being, diversity, and adaptability (39). In the context of RTW and psychological empowerment, SHRM plays a pivotal role in creating structured, inclusive, and sustainable reintegration practices. For example, Philpot and Gavrilova Aguilar (14) noted that effective SHRM ensures organizations develop and implement RTW programs that are not only legally compliant but also psychologically and socially supportive. This includes customized reintegration plans, mental health support, workplace accommodations, and communication strategies designed to rebuild trust and motivation among returning employees (40). A growing body of literature supports this link. For instance, Krause et al. (41) highlight that organizations with strategic HR frameworks are more successful in reducing long-term absenteeism and supporting reintegration through tailored RTW interventions. Similarly, Munir et al. (42) argue that strategic practices—such as flexibility, health promotion, and career development—create a positive psychological empowerment that encourages employees to re-engage with their roles. van Duijn et al. (18) found that companies adopting SHRM-driven RTW strategies reported higher return rates, shorter recovery-to-work durations, and improved employee retention. Furthermore, SHRM reinforces organizational readiness to support returning employees not only structurally but also emotionally. By promoting a culture of inclusion, participation, and continuous dialogue, SHRM fosters employee psychological empowerment and well-being, both of which are essential to successful RTW outcomes. Moreover, most SHRM studies focus on employee performance, engagement, or retention, with limited attention given to reintegration after an absence, especially in sectors such as healthcare, education, and manufacturing, where workforce continuity is critical. Additionally, evidence is scarce in emerging economies, where formal RTW programs and strategic human resource (HR) practices are still evolving. This study addresses these gaps by exploring the direct influence of SHRM on RTW and psychological empowerment outcomes, thereby contributing to a more strategic and integrated understanding of workforce reintegration. Therefore, the present study proposed the following hypotheses.

H6: Strategic human resource management has a significant and positive impact on the return to work

H7: Strategic human resource management practice has a significant and positive impact on psychological empowerment

### 2.4 Psychological empowerment and return to work

Psychological empowerment refers to an individual's fundamental motivation manifested through a sense of competence, meaning, impact, and self-determination at work (72). Practically, empowered employees act more confidently in their potential capability to perform tasks, overcome challenges, and contribute widely to sustain competitive advantages and organizational goals. In the context of RTW, psychological empowerment plays a broader role in rebuilding the control and self-efficacy that may be diminished due to illness, injury, and/or mental disturbances (73). Empirically, several existing studies examined and supported the significant positive impact of psychological empowerment on SHRM practices. For example, Zhang and Wang (74) found that SHRM practices promoting flexibility and inclusion are significantly supported by psychological empowerment across diverse work environments. Similarly, Cheng and Zou (75) indicate that empowerment-oriented HR practices—such as establishing performance appraisals and providing training opportunities—maximize employees' sense of autonomy and competence. Additionally, Yakut and Kara (76) argue that empowerment serves as a mediating mechanism through which SHRM shapes enhanced organizational outcomes, including proactive work behaviors and job satisfaction. Therefore, the key role of psychological empowerment in cultivating SHRM remains underexplored in RTW models, particularly in emerging economies or post-pandemic work environments, where employee autonomy and resilience are more crucial than ever. Limited empirical studies have empirically examined and validated the pathway from psychological empowerment to SHRM and then to RTW outcomes within an integrated framework. The present study addresses the above arguments by exploring how psychological empowerment impacts SHRM and, in turn, how empowerment affects RTW, offering new insights into the human-centered dimensions of strategic reintegration planning. Therefore, the following hypothesis was proposed.

H8: Psychological empowerment has a significant and positive impact on return to work

## 3 Method

### 3.1 Sampling and target population

A stratified sampling technique was employed to collect data from 370 employees who had successfully returned to work after any injury and/or illness in public, private, and semi-public organizations in Jeddah, Saudi Arabia. The data for the present study were collected from February to May 2025, during the post-COVID era. However, the present study did not explicitly consider COVID-19 as a specific factor influencing RTW of injured and/or ill employees related to work absence. However, a cross-sectional research design limits causal inference, as it does not account for participants from different sectors, including healthcare, education, manufacturing, and services. For more validity, the present study performed “Harman’s single-factor” test to check for potential common method bias, and the results indicated a 27% threshold, confirming the absence of common method bias. Thus, the inclusion criteria required participants to be full-time employees aged 18 or older, with at least 6 months of tenure in their current organization, and to have successfully RTW after illness or injury. However, male respondents accounted for 59.5% of the sample, while the majority were in the 26–35 years age range (37.8%). Most participants held either a Bachelor's degree (45.9%) or worked in the public sector (48.6%), with many also working as mid-level managers (48.6%). Therefore, a significant portion had been with their organizations for 4–7 years (37.8%). Finally, 29.7% of respondents had an absence of 1–3 months, 27.0% had an absence of 4–6 months, and 21.6% had an absence of more than 6 months. Thus, the respondents' demographic characteristics are presented in Table 1.

**Table 1:** Demographic characteristics.

Demographic item	Category	Frequency (n)	Percentage (%)
Gender	Male	220	59.5
	Female	150	40.5
Age	18–25 years	60	16.2
	26–35 years	140	37.8
	36–45 years	110	29.7
	Above 45 years	60	16.2
Educational Qualification	Bachelor degree	170	45.9
	Masters degree	150	40.5
	PhD	30	8.1
	Others	20	5.4
Sector	Public	180	48.6
	Private	160	43.2
	Semi-government	30	8.1
Job Level	Entry-level	90	24.3
	Mid-level	180	48.6
	Senior-level	70	18.9
	Top Management	30	8.1
Length of Service	<1 year	40	10.8
	1 years	120	32.4
	4–7 years	140	37.8
	>8 years	70	18.9
Duration of Absence	<1 month	80	21.6
	1–3 months	110	29.7
	4–6 months	100	27
	>6 months	80	21.6

### 3.2 Development of measurements

The measurement items were developed using validated measurement scales adapted from existing studies. For instance, the measurement items for the four dimensions of psychological empowerment (i.e., meaning, competence, self-determination, and impact) were adapted from the validated scale developed by Chiang and Hsieh (27). Items measuring organizational hierarchy culture were adapted from Zeb et al. (43). The construct of SHRM practices was adapted from Zehir et al. (15). Finally, the five dimensions of RTW (i.e., prepared for action, contemplation, uncertain maintenance, proactive maintenance, and maintenance) were measured using items adapted from Franche et al. (1), a widely recognized scale in occupational health research focused on work reintegration stages.

A 5-point Likert scale ranging from 1 (“strongly disagree”) to 5 (“strongly agree”) was used to validate the items (44). Thus, the main questionnaire consisted of two sections: (1) the demographic characteristics of the participants and (2) measurement items of all constructs. Before collecting the data, the questionnaire was pre-tested by field experts (two from academia and two from industry), and minor amendments were made based on their feedback. After that, this study conducted a pilot study from (*n* = 35) to ensure the clarity, relevance, and cultural appropriateness of the main questionnaire. However, the questionnaire was distributed electronically via email and WhatsApp invitations, and physically in selected organizations via paper-based forms, to improve response rates. Respondents were assured of confidentiality and anonymity to encourage honest and unbiased responses. The survey was made available in both English and Arabic to accommodate linguistic preferences and enhance comprehension.

### 3.3 Measurement

All measurement items for the present study were evaluated using Cronbach's Alpha (*α*), composite reliability (CR), and average variance extracted (AVE) to assess convergent validity (45). The threshold value for all measurement items' loadings was reported as above 0.7 (46). In particular, higher-order model analysis was employed to capture the multidimensional nature of psychological empowerment, potentially. RTW was conceptualized as a second-order reflective construct, as suggested by Franche et al. (1). This approach enables a more comprehensive representation of complex theoretical concepts by aggregating their distinct yet interrelated dimensions into a unified, higher-order structure. Notably, the factor loadings for the dimensions of psychological empowerment ranged from 0.659 to 0.918, perceived organizational support from 0.616 to 0.837, organizational hierarchy culture from 0.641 to 0.818, SHRM from 0.603 to 0.821, and RTW from 0.632 to 0.899. In this view, CR values for all key constructs reported above the acceptance level of 0.60. To further validate the complex model, the present study also tested the discriminant validity of the AVEs presented in Table 2. Thus, factor loading of all items and *α* and CR values of all key constructs and their dimensions are presented in Table 3.

**Table 2:** HTMT discriminant validity.

Constructs	COM	CON	IM	ME	OE	OHC	PA	PC	PM	POS	RTW	SD	SHRM	UM
COM														
CON	0.503													
IM	0.450	0.208												
ME	0.800	0.394	0.629											
OE	0.148	0.509	0.764	0.710										
OHC	0.415	0.539	0.391	0.427	0.540									
PA	0.452	0.835	0.591	0.502	0.614	0.569								
PC	0.433	0.839	0.049	0.243	0.393	0.364	0.455							
PM	0.297	0.544	0.595	0.367	0.486	0.563	0.712	0.324						
POS	0.412	0.230	0.679	0.871	0.659	0.318	0.488	0.101	0.554					
RTW	0.531	0.799	0.476	0.439	0.611	0.610	0.726	0.776	0.720	0.411				
SD	0.809	0.509	0.287	0.649	0.745	0.347	0.411	0.473	0.298	0.279	0.495			
SHRM	0.407	0.616	0.673	0.397	0.563	0.619	0.781	0.304	0.735	0.511	0.752	0.343		
UM	0.541	0.649	0.632	0.321	0.579	0.580	0.788	0.483	0.788	0.372	0.709	0.401	0.818	

**Table 3:** Measurement items.

Construct	Item Code	Loading	*α*	CR	AVE
Perceived organizational support			0.720	0.897	0.515
	POS1	0.837			
	POS2	0.779			
	POS3	0.632			
	POS4	0.616			
	POS5	0.662			
Psychology empowerment			0.705	0.733	0.532
Meaning			0.680	0.760	0.520
	ME1	0.659			
	ME2	0.751			
	ME3	0.731			
Competence			0.750	0.820	0.600
	COM1	0.727			
	COM2	0.795			
	COM3	0.777			
Self-determination			0.850	0.910	0.841
	SD1	0.895			
	SD2	0.876			
Impact			0.910	0.930	0.861
	IM1	0.914			
	IM2	0.918			
	IM3	0.830			
Organizational hierarchy culture			0.824	0.876	0.574
	OHC1	0.641			
	OHC2	0.664			
	OHC3	0.807			
	OHC4	0.818			
Strategic human resource management			0.775	0.843	0.523
	SHRM1	0.725			
	SHRM2	0.821			
	SHRM3	0.722			
	SHRM4	0.603			
	SHRM5	0.786			
	SHRM6	0.808			
	SHRM7	0.745			
	SHRM8	0.730			
	SHRM9	0.682			
Return to work			0.736	0.893	0.757
Uncertain Maintenance			0.884	0.914	0.788
	UM1	0.899			
	UM2	0.821			
	UM3	0.813			
	UM4	0.844			
Prepared for action			0.741	0.824	0.547
	PA1	0.632			
	PA2	0.623			
	PA3	0.799			
	PA4	0.724			
	PA5	0.695			
Contemplation			0.793	0.850	0.661
	CON1	0.768			
	CON2	0.745			
	CON3	0.816			
Precontemplation			0.868	0.907	0.758
	PC1	0.869			
	PC2	0.860			
	PC3	0.882			
	PC4	0.835			
Proactive Maintenance			0.802	0.863	0.614
	PM1	0.779			
	PM2	0.743			
	PM3	0.785			
	PM4	0.683			
	PM5	0.672			

## 4 Data analysis and results

Structural equation modeling (SEM) was applied to validate the direct and indirect hypotheses proposed in this study. SEM approaches involve assessing the evaluation of the study's complex model and estimating the structural coefficient path (44). This statistical modeling method is widely preferred in management and social science studies to validate the model's reliability using the data (46). Therefore, two approaches [i.e., covariance-based structural equation modeling (CB-SEM) and partial least squares structural equation modeling (PLS-SEM)] have been applied in structural equation modeling (SEM) via Smart PLS software. Thus, the present study employed PLS-SEM because it has strong potential to highlight the complex relationships among constructs, justify theoretical approaches, and estimate path coefficients with reasonable accuracy (77). The PLS approaches evaluate the model using two broad approaches [i.e., a structural model (outer) and a measurement model (inner)] that draw paths among the latent variables. Therefore, in the present study, psychological empowerment and RTW were tested using higher-order model techniques. Hence, the results of the direct and indirect hypotheses are reported in Table 4.

**Table 4:** Path coefficient.

Paths	Beta	*T* statistics	*P* values
Perceived Organizational Support → Return to Work	0.005	0.072	0.943
Perceived Organizational Support → Strategic Human Resource Management	0.175	2.301	0.021
Perceived organizational support → psychology empowerment	0.183	2.718	0.023
Organizational Hierarchy Culture → Return to Work	0.234	3.124	0.002
Organizational Hierarchy Culture → psychology empowerment	0.439	7.170	0.000
Strategic Human Resource Management → Return to Work	0.453	6.668	0.000
Strategic Human Resource Management → empowerment	0.247	3.127	0.002
Psychology empowerment → Return to Work	0.153	2.100	0.036

**Figure 1:**
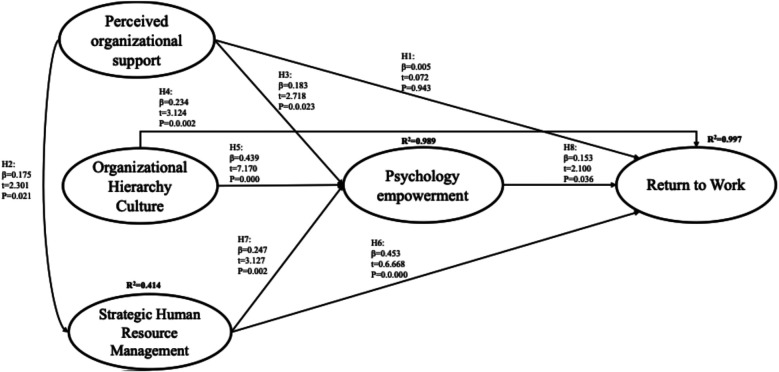
Structural model of return-to-work.

In this study, path coefficients, t-statistics, and *p*-values were employed to validate the hypothesized relationships among the key constructs. For instance, Hypothesis (H1) indicates that the perceived organizational support has an insignificant impact on the RTW (*t*-value = 0.072; *p* > 0.05), suggesting that H2 is not supported. On the other hand, perceived organizational support has a significant impact on SHRM (t-value = 2.301; *p* < 0.05) and psychological empowerment (*t*-value = 2.718; *p* < 0.05), thereby supporting H2 and H3. Additionally, the present study confirmed a significant impact of organizational hierarchy culture on RTW (*t*-value = 3.124; *p* < 0.05) and psychological empowerment (*t*-value = 7.170; *p* < 0.01), thereby supporting H4 and H5. Notably, the present study also tested and confirmed the direct and significant impact of SHRM on return-to-work (RTW (*t*-value = 6.668; *p* < 0.001) and psychological empowerment (*t*-value = 3.127; *p* < 0.05); therefore, H6 and H7 are supported. Finally, psychological empowerment has a significant impact on RTW (*t*-value = 2.100; *p* < 0.05), supporting H8. In summary, the findings from the present study suggest that SHRM acts as a central facilitator of successful RTW, influenced by organizational culture, perceived organizational support, and employee empowerment. The non-significant impact of perceived organizational support on RTW highlights a research gap and underscores the need for future studies to explore possible mediators or contextual moderators (e.g., trust, leadership style) that shape this relationship in the Saudi Arabian context. Therefore, overall hypothetical results are presented in Table 4 and Figure 1.

## 5 Mediating hypotheses

Table 4 presents the mediating role of SHRM between perceived organizational support, psychological empowerment, organizational hierarchy culture, and RTW. The statistical analysis confirmed a significant mediating role of SHRM. Thus, the overall results are presented below.

H8 demonstrates that SHRM mediates the relationship between perceived organizational support and RTW (*t*-value = 2.178; *p* < 0.05), thereby supporting H8. Furthermore, psychological empowerment indirectly impacts RTW mediated by the SHRM (*t*-value = 2.894; *p* < 0.01), confirming that H9 is supported. Finally, SHRM mediates the relationship between organizational hierarchy culture and RTW (*t*-value = 5.632; *p* < 0.001), thus H10 is supported. Therefore, the overall results of the mediating hypotheses are presented in Table 5.

**Table 5:** Mediating hypotheses.

Indirect paths	Beta value	*T* value	*P* values
Psychology empowerment → Strategic Human Resource Management → Return to Work	0.130	2.894	0.004
Perceived organizational support → Strategic Human Resource Management → to Work	0.091	2.178	0.029
Organizational Hierarchy Culture → Human Resource Management → Return to Work	0.226	5.632	0.000

## 6 Discussion

The findings from the present study provide meaningful insights into the psychological and organizational factors that influence RTW outcomes, particularly in the context of Saudi Arabia. The results demonstrated that psychological empowerment and organizational hierarchy culture have significant positive effects on RTW and shared health and resource management, confirming that both structural and human-centric aspects of the workplace environment play an essential role in facilitating employee reintegration after a period of absence. Surprisingly, the present study found that perceived organizational support had an insignificant impact on RTW.

First, perceived organizational support did not have a statistically significant direct impact on RTW. This result is consistent with the past studies. For example, Sitorus et al. (47) found no relationship between perceived organizational support and RTW. On the other hand, Shaw et al. (30) witnessed that contextual and cultural differences usually cause a lack of organizational support in RTW for employees diagnosed with mental and/or physical illness. Notably, in highly power-distance or hierarchical Arab cultures, such as Saudi Arabia, employees may not view perceived relational or emotional support as a sufficient driver for RTW unless it is accompanied by formalized HR policies and procedural clarity (18). On the other hand, the present study confirmed a significant direct relationship between perceived organizational support and SHRM, as well as between perceived organizational support and psychological empowerment. As highlighted by Farooq and Javid (78), in such Arab contexts, organizational support tends to be interpreted as symbolic unless it is reinforced through institutional mechanisms. This aligns with the present study's finding that perceived organizational support becomes more impactful when delivered through SHRM practices. The integration of organizational support and SHRM interventions, such as customized RTW plans, inclusive workplace policies, and training for line managers, contributes to sustainable reintegration (79). Consequently, the insignificant direct impact observed may reflect a gap between perceived support and its practical implementation, reinforcing the idea that supportive climates must be translated into tangible, strategic actions to effectively enhance RTW outcomes for employees who are mentally and/or physically ill.

Second, the present study revealed a significant direct impact of organizational hierarchy culture on RTW and psychological empowerment. This aligns with the findings of existing studies, which suggest that organizational cultures emphasizing control and stability may enhance predictability and reduce the psychological burden of reintegration. For example, an empirical study by Jetha et al. (48) examined and concluded that the organizational hierarchy has a significant influence on RTW. In this view, Yakut & Kara (49) indicated that the organizational hierarchy significantly predetermines SHRM practices. On the other hand, the findings of the present study are particularly relevant in the context of Saudi Arabia's organizational and cultural setting, characterized by high power distance, centralized decision-making, and formalized workplace structures (50).

Contrary to the typical Western organizational assumption that hierarchical cultures suppress empowerment (51, 52), hierarchical structures in Saudi Arabia may foster a sense of psychological safety and clarity (53), which, in turn, contributes to feelings of empowerment. As argued by Tlaiss (54), structured hierarchies in Arab workplaces provide employees with clear role expectations, defined career paths, and procedural consistency, which enhance their sense of competence and impact, key dimensions of psychological empowerment. Further supporting this, Ayyash et al. (55) outlined that in Saudi Arabian organizations, especially in the public sector, employees often associate empowerment not with autonomy in a Western sense but with recognition, status, and structured guidance. Employees who understand the chain of command and receive clear directives are more likely to feel confident and effective in their roles, fulfilling the self-determination and meaning aspects of empowerment as conceptualized by Yakut & Kara (49). Therefore, the significant relationship observed in this study aligns with culturally contingent perspectives of empowerment. It suggests that in high power-distance societies, such as Saudi Arabia, empowerment can be effectively nurtured through organizational structures that emphasize stability, procedural clarity, and formal support rather than through flat or decentralized models common in Western contexts.

Third, the findings of the present study demonstrate that SHRM has a substantial and statistically significant impact on RTW and a moderate yet significant impact on psychological empowerment. These results highlight the crucial and facilitative role that strategically aligned HR practices play in employee reintegration and empowerment, particularly in structured, high-power-distance organizational cultures such as those found in Saudi Arabia. In Saudi Arabia, SHRM practices such as structured onboarding, continuous professional development, performance management, and well-defined reintegration policies significantly contribute to employees' successful RTW. As noted by Tlaiss & Al Waqfi (56), effective SHRM in the Arab region is characterized by centralized planning, robust policy enforcement, and respect for hierarchy, which align well with the national culture's emphasis on authority and stability. In hierarchy-oriented cultures, SHRM is often implemented through formalized structures, top-down policy dissemination, and strict performance metrics. While Philpot & Gavrilova Aguilar (14) noted that such a structure can bring discipline and clarity to HR practices, it can also limit the adaptability and innovativeness that SHRM requires, especially when addressing dynamic issues such as workforce diversity, remote work, or RTW processes. The success of SHRM in such cultures depends on how well hierarchical values (e.g., consistency, standardization) are balanced with strategic needs (e.g., flexibility, employee empowerment) (57). Additionally, the significant relationship between SHRM and psychological empowerment suggests that when HR practices are strategically tailored to individual and organizational goals, they not only facilitate operational efficiency but also enhance employees' sense of competence, autonomy, and meaning. As indicated by Zehir et al. (15) and supported in the Arab context by Alsarhan et al. (58), SHRM frameworks that promote clear communication, regular feedback, and empowerment-oriented training can significantly uplift employee motivation and ownership. In cultures like Saudi Arabia, where formal authority is highly valued, empowerment often arises not only from autonomy in decision-making but also from structured guidance, recognition, and professional development opportunities embedded within HR systems. Therefore, SHRM acts as a conduit between organizational structure and employee agency, enabling individuals to regain confidence, perform effectively, and perceive their return as meaningful and supported. In summary, these findings from the present study validate the assertion that SHRM not only facilitates functional reintegration and RTW but also nurtures psychological reintegration, reinforcing the need for organizations to invest in strategic, culturally aligned human resource management (HRM) systems to maximize post-absence workforce reintegration. Importantly, SHRM supports the development and implementation of policies to support employees as they transition back to work. In contexts where clarity, formal procedures, and defined roles dominate, employees may feel more secure and guided during their reintegration process (6). Such an environment likely provides standardized protocols, consistent communication, and accountability mechanisms that reduce uncertainty, thereby encouraging timely and confident returns.

Fourth, the present study demonstrated a significant impact of psychological empowerment on RTW, suggesting that employees who feel autonomous, competent, and impactful are more likely to overcome RTW barriers. Thus, the findings of the present study align with previous studies. For instance, Rahi (59) confirmed a significant impact of psychological empowerment on RTW and work engagement. Accordingly, a recent study by Monje-Amor et al. (60) demonstrated that psychological empowerment is significantly associated with a solid commitment to RTW goals, particularly in psychologically demanding professions such as healthcare and education. Empowerment fosters employees' sense of agency and enhances internal motivation, both of which are critical for navigating the emotional and physical challenges often associated with returning to the workplace (24). Green et al. (61) found that when employees felt autonomous and competent, they reported lower stress and higher satisfaction during the RTW transition. Another study by Hoefsmit et al. (62) found that empowerment enhanced the willingness of employees with disabilities or injuries to RTW and minimize anxiety associated with resuming their responsibilities. Although psychological empowerment has been acknowledged widely in scholarly work as a critical psychological driver of work outcomes, limited past studies explicitly link it to RTW, specifically in contexts involving mental and physical recovery. Overall, the findings from the present study suggest that psychological readiness is a crucial determinant of successful work resumption, particularly in contexts that require the adjustment or accommodation of employees with disabilities and/or illnesses.

Finally, the present study also validated the mediating role of SHRM between perceived organizational support, psychological empowerment, organizational hierarchy culture, and RTW. The mediation analysis exhibits that SHRM plays an essential role in translating the impact of these predictors into actual RTW outcomes. Each of the three antecedents (i.e., psychological empowerment, perceived organizational support, and organizational hierarchy culture) had a significant indirect effect on RTW through SHRM. These findings strengthen the notion that while organizational characteristics and employee attitudes are foundational, it is the strategic implementation of HR practices that operationalizes these elements into structured support mechanisms. For instance, Zehir et al. (15) stated that empowerment may encourage engagement, but it is through SHRM that empowerment is nurtured via development, training, and inclusive policies. Similarly, Shrestha & Prajapati (39) noted that a supportive climate must be reflected in strategic initiatives such as return-to-work plans, wellness programs, and open communication channels. In the same vein, organizational hierarchy culture must be embodied in consistent HR procedures to truly benefit reintegration outcomes truly.

In summary, the findings from the present study suggest the need for a more integrated framework of RTW that accounts for the relationship between organizational hierarchy culture, employee perceptions, and Strategic Human Resource Management (SHRM) strategies. While prior literature often isolates these variables, this study demonstrates their interconnectedness in shaping RTW success. This contributes to the growing body of literature that emphasizes multi-level influences on workplace behavior and highlights the importance of aligning individual, cultural, and strategic dimensions.

### 6.1 Managerial and practical implications

The findings of this study offer several important managerial and practical implications for organizational leaders, human resource professionals, and policy designers seeking to enhance RTW outcomes in structured workplace environments. The current study aims to raise awareness among public and private organizations in Saudi Arabia about the importance of developing a strategic, practical plan to facilitate RTW for sick and/or disabled employees. Notably, the current study's findings suggest the cultural underpinnings of RTW in Saudi Arabian organizations, particularly the influence of high power distance and hierarchical structures on employee perceptions and empowerment.

First, the role of perceived organizational support in influencing RTW emphasizes the importance of cultivating a supportive work environment. Managers should go beyond providing administrative assistance and focus on expressing genuine empathy, care, and appreciation for employees during their transition to RTW. This could include regular check-ins, personalized accommodations, wellness initiatives, and peer support systems. Second, the significant contribution of psychological empowerment to RTW and SHRM outcomes suggests that empowering employees with responsibility, autonomy, and meaningful work may enhance their readiness for RTW. Thus, HR departments should design interventions that enhance self-efficacy, such as mentoring, job redesign, participative decision-making, and access to learning resources. Empowered employees are more resilient and confident in managing challenges during reintegration, making empowerment a strategic tool for return-to-work success (62). Third, the significant impact of organizational hierarchy culture on RTW and SHRM underscores the value of a structured, rule-driven work environment. Thus, managers should ensure that documented procedures govern reintegration processes, clear policies are in place, and standardized communication protocols are established. Such structural clarity reduces ambiguity and fosters psychological security among returning employees (63). Therefore, organizations should formalize RTW guidelines and incorporate them into employee handbooks, orientation programs, and management training to ensure consistent and supportive practices (23). Notably, SHRM's significant role demonstrates that the effectiveness of organizational culture, empowerment, and support depends on how well it is translated into HR policies and practices. In such contexts, fostering a culture of compassion can reinforce employees' trust and motivation to re-engage with their work roles (64). By integrating cultural considerations with strategic HR practices, organizations can enhance RTW outcomes and overall employee well-being. On the other hand, managers must adopt a strategic lens in designing HR initiatives that align with long-term reintegration goals. This includes developing comprehensive RTW programs that combine policy structure, emotional support, and individualized reintegration plans. Moreover, SHRM practices should be embedded within the broader talent management and organizational development frameworks to ensure sustainability (32).

In sum, for practitioners in the Saudi context, these findings are particularly relevant as many organizations are undergoing reforms in line with Vision 2030. Vision 2030 serves as a strategic plan for Saudi Arabia to achieve social, environmental, and economic transformation through organizational reforms, efficiency, emphasizing modernization, and employee-centric practices (65). It encourages organizations to adopt progressive workplace policies through SHRM practices to support workforce engagement and empowerment in private and public organizations. This study strengthens the argument that effective reintegration is not solely a compliance function but a strategic, human-centered process that requires coordination across departments and levels of leadership. Furthermore, managers should focus on creating an environment that combines clarity (through culture), care (through support), and confidence (through empowerment)—all embedded and reinforced through strategic HRM systems. In this view, organizations may significantly enhance the long-term retention and reintegration experience of employees returning after absence due to mental and/or physical illness.

## 7 Limitations

The present study, which offers valuable insights into the organizational, psychological, hierarchical cultural, and SHRM practices that influence RTW, is subject to several limitations that future researchers should acknowledge.

First, the research employed a stratified sampling technique, which limits the ability to infer causality among the variables under study. Although significant associations were identified, longitudinal studies are needed to determine the temporal sequence of influence, particularly during employee reintegration. Future research could adopt a longitudinal approach to examine how factors such as organizational support and empowerment evolve across the pre-return, reintegration, and post-return phases. Second, the study relied on self-reported data collected via structured questionnaires, which may be subject to common-method bias and social desirability bias. While statistical techniques were applied to minimize bias, future studies could incorporate multi-source data collection methods, such as combining employee self-assessments with supervisor evaluations or organizational performance records, to improve the robustness of findings. Third, the research was conducted within the socio-cultural context of Saudi Arabia, which is characterized by hierarchical workplace norms and collectivist values. While this provides deep contextual insights, it may limit the generalizability of results to other regions with different organizational cultures and employee expectations. Comparative studies across different countries or sectors can offer a broader understanding of how cultural variables moderate the RTW process. Another limitation is the exclusion of potential mediating and/or moderating variables that could further explore the dynamic relationships among constructs. For instance, trust in management, leadership style, or mental health status may influence how perceived support or SHRM practices impact RTW outcomes. Future research should explore these variables using more complex models, such as moderated mediation or multigroup analysis, to capture more nuanced structural relationships.

## Data Availability

The raw data supporting the conclusions of this article will be made available by the authors, without undue reservation.
